# Dual Piezoelectric Energy Investing and Harvesting Interface for High-Voltage Input

**DOI:** 10.3390/s21072357

**Published:** 2021-03-28

**Authors:** Muhammad Bilawal Khan, Hassan Saif, Kyoungho Lee, Yoonmyung Lee

**Affiliations:** 1 Department of Electrical and Computer Engineering, Sungkyunkwan University, Suwon 16419, Korea; bilawal786@skku.edu; 2 Department of Electrical Engineering, National University of Computer and Emerging Sciences, Islamabad 44000, Pakistan; hassan.saif@nu.edu.pk; 3 Korea Electrotechnology Research Institute, Changwon 51543, Korea; khlee93@keri.re.kr

**Keywords:** energy harvester, energy investment, harvesting interface, high-voltage harvesting, partial electric charge extraction (PECE), piezoelectric generator, PZT

## Abstract

A novel harvesting interface for multiple piezoelectric transducers (PZTs) is proposed for high-voltage energy harvesting. Pre-biasing a PZT prior to its mechanical deformation increases its damping force, resulting in higher energy extraction. Unlike the conventional harvesters where a PZT-generated output is assumed to be continuous sinusoidal and output polarity is assumed to be alternating every cycle, PZT-generated output from human motion is expected to be random. Therefore, in the proposed approach, energy is invested to the PZT only when PZT deformation is detected. Upon the motion detection, energy stored at a storage capacitor (*C_STOR_*) from earlier energy harvesting cycle is invested to pre-bias PZT, enhancing energy extraction. The harvested energy is transferred to back *C_STOR_* for energy investment on the next cycle and then excess energy is transferred to the battery. In addition, partial electric charge extraction (PECE) is adapted to extract a partial amount of charges from the PZT every time its voltage approaches the process limit of 40 V. Simulations with 0.35 µm BCD process show 7.61× (with PECE only) and 8.38× (with PECE and energy investment) improvement compared to the conventional rectifier-based harvesting scheme Proposed harvesting interface successfully harvests energy from excitations with open-circuit voltages up to 100 V.

## 1. Introduction

With ever-increasing interest in wearable electronics, many smart devices, such as smart watches, earphones, rings, and glasses, have been developed. Moreover, many devices for healthcare, such as human body posture, heart rate, blood pressure, sugar level, and wound monitors, have also been developed. These devices are usually powered with lightweight batteries having low capacity that require frequent battery re-charging or replacement, which is undesirable and sometimes difficult in the case of implantable devices. To achieve energy autonomy, widely investigated energy harvesting sources include RF [1,2,3,4], light [5], heat [6], and vibrations [7,8,9]. Among these sources, vibration energy has been extensively investigated due to the abundance of vibrations in the ambient environment. For this purpose, various electromechanical transducers, such as electromagnetic [10], triboelectric [11], and piezoelectric [12] transducers, are usually utilized.

Due to their light weight, flexibility, high energy density, and easy integration, piezoelectric transducers (PZTs) are widely used for kinetic energy harvesting. Multiple PZTs can be easily attached to human body joints, such as elbows or knees, to take advantage of vibrations resulting from human motion. As these vibrations are irregular, PZT generates irregular output with aperiodic pulses, which require a harvesting interface circuit for energy harvesting. Although many harvesting interface designs have been proposed to harvest energy from a single PZT [13,14,15,16,17], there has not been much work focused on harvesting energy from multiple PZTs attached to the human body. As energy generated by a single PZT could be insufficient to power wearable electronics, this work focuses on maximizing energy extraction from multiple PZTs.

**Figure 1:**
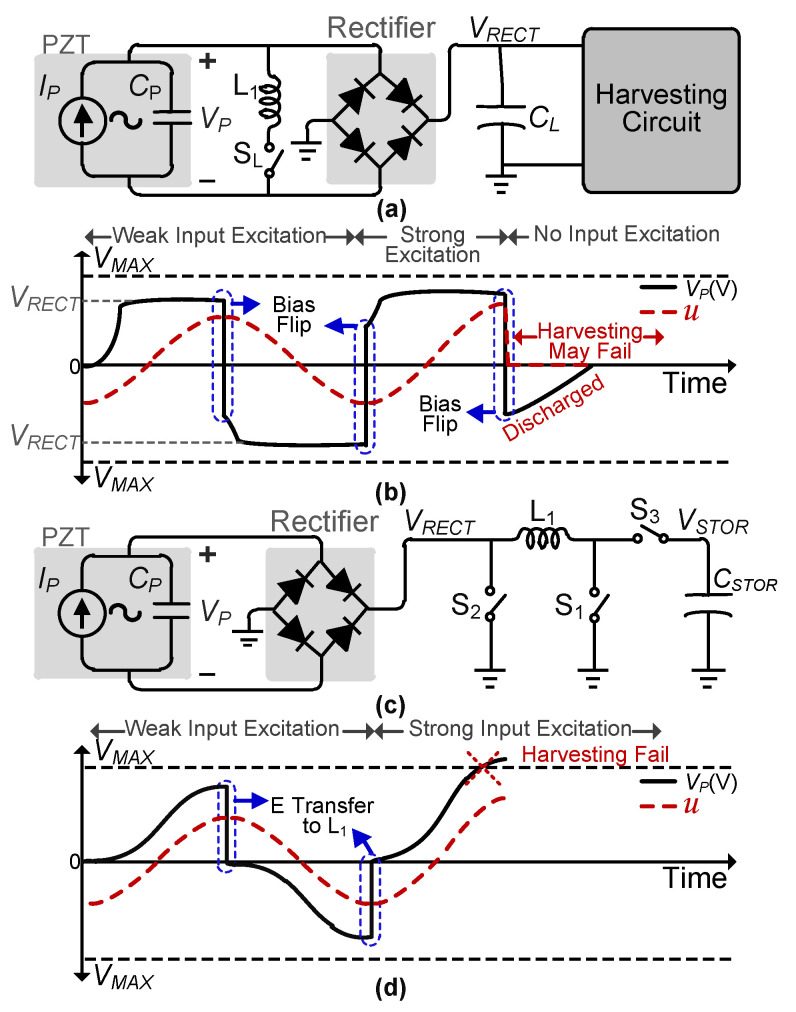
(**a**) SSHI-based harvesting interface circuit, (**b**) its operation waveform with different input excitations, (**c**) SECE-based harvesting interface circuit, and (**d**) its operation waveform with different input excitation strengths.

Fabrication and characterization of multiple vibration transducers-based systems have been done in some of the prior works [18,19,20]. However, they only utilize simple circuits with discrete components or commercially available harvesting circuits to evaluate harvesting performance and did not present any optimized integrated circuit design for multiple transducers. Several investigations have been done on mathematical modelling of piezoelectric harvesters [21,22,23]. Cantilever beam configuration is the most commonly used configuration for piezoelectric energy harvesting. In this configuration, one end of PZT is fixed and a mass is attached to the other end. This can be represented as an electromechanical model [24]. In circuit domain, the PZT model can be simplified as a current source (PZT-generated current, *I_P_*) in parallel with the PZT’s internal capacitance (*C_P_*) [25], as shown in Figure 1a. The electromechanical model is utilized if accurate results in combination with interface circuits are required. If the focus lies exclusively on the designing interface circuit and resonance/feedback characteristics are not important, the simplified circuit (uncoupled model) can be used [26,27]. As focus of the proposed work is on harvesting circuit design, our analysis will be based on this electrical model of PZT. 

When mechanical displacement (*u*) is applied to a PZT, *I_P_* is generated, which charges *C_P_*, and an output voltage (*V_P_*) is developed across *C_P_*. The generated charge (*Q_P_*) is proportional to the physical deformation applied to the PZT. Therefore, for a given amount of physical deformation, *Q_P_* remains the same even if the load capacitance (*C_L_*) seen by the PZT changes [28]. PZT-generated energy (*E_P_*) from a single deformation can be written as follows:

(1) $$E_{P}=\;\frac{1}{2}\left(C_{P}+C_{L}\right)V_{P}^{2}=\frac{1}{2}\frac{Q_{P}^{2}}{C_{P}+C_{L}}$$

where *V_P_* is the final (peak) PZT voltage at the maximum/minimum *u*. This implies that for any *u* applied to the PZT and generated *Q_P_*, *E_P_* is inversely proportional to load capacitance seen by the PZT. A smaller load capacitance allows the faster development of *V_P_* and therefore makes the PZT damping force stronger [29,30]. Hence, for the same amount of *u*, more energy is required; therefore, more mechanical energy is converted to electrical energy. This means that to capitalize on the extractable energy from the PZT, the load capacitance seen by the PZT should be minimized [28].

Numerous harvesting interface circuits have been proposed [17,25,27,30,31,32,33,34] to efficiently harvest energy generated by vibrations applied to the PZT. Most of these prior works focus on harvesting energy from continuous (sinusoidal) or shock (decaying sinusoidal) excitations applied to the PZT. Some prior works, such as [21,22,23], rely on cantilever-beam configuration for measurement results for different operation frequencies (resonance and off-resonance), which is not applicable to the proposed work, which focuses on energy harvesting from random input pulses generated by human motion. Synchronous switch harvesting on inductor (SSHI) [17,25,31,32] is one of the most widely adopted piezoelectric energy-harvesting interface designs. A simplified circuit diagram of an SSHI-based harvesting interface is shown in Figure 1a. A rectifier is essential to convert PZT-generated alternating output voltage to the rectified *V_RECT_*. Initially, input excitations applied to the PZT are assumed to be continuous, as shown in Figure 1b. The polarity of *V_P_* is flipped (using an inductor) at every half cycle of *I_P_* (maximum/minimum of *u*) to extract more energy from the PZT in the next half cycle. This means that the PZT voltage will be already at a higher value (behaving as pre-biased) as the next half cycle starts, which strengthens the damping force [30], and more energy is extracted from the PZT. However, these types of harvesters are not optimized for mechanical inactivity periods, which might be possible when the human body is at rest. During an inactive period, *C_P_* will discharge (depending on the circuit/PZT resistance), which could compromise the harvesting interface circuit functionality. In addition, energy (*E_PZT_*) extraction from the PZT will decrease during the next deformation as *C_P_* would need to be charged from a lower voltage as the deformation starts. Therefore, bias-flipping at every maximum/minimum of u is not suitable for irregular human motion.

One solution to avoid a potential bias-flip circuit failure due to random input is a synchronous electric charge extraction (SECE) technique [27,33,34], in which bias-flip is not performed. In SECE-based PZT harvesting interface circuits [27,33,35], which can be simplified as shown in Figure 1c, *V_P_* is increased to its peak before starting a complete electric charge extraction from *C_P_* using an inductor. This means that the PZT-generated voltage starts from zero (without pre-bias) at the beginning of each deformation, which results in a lower damping force on the PZT [30] and hence limited energy extraction in each harvesting cycle. A variation of SECE, called delayed-SECE [36], aims at increasing energy extraction by sending part of the generated-energy back to the PZT at the end of one half-cycle of *I_P_* with the help of an inductor. This way, *V_P_* is already high at the start of the next half-cycle of *I_P_*. However, circuit functionality (in case of random input) is compromised by doing bias-flip like operation (after peak detection) at the end of each deformation. Therefore, energy investment at the correct instant is critical for maximum energy extraction, and an optimized circuit needs to be designed for this purpose. 

Moreover, as harvesting sources (piezo/tribo-electric transducers) are improved, higher energy can be extracted with higher input voltage [28,37,38,39,40]. Designing a circuit to harvest energy from these high-input voltages is a non-trivial challenge, and it becomes even more challenging when these output voltages can exceed the process limit. In SSHI-based harvesting interfaces, a large load (buffer) capacitor (*C_L_*) is required at the output of the rectifier, as shown in Figure 1a. Typically, *C_P_* of PZTs used for energy harvesting is in the order of 100 s of pF to 10 s of nF (in [17,27,28,29,31,32]). Whereas, typical value of *C_L_* used by SSHI-based harvesters is in the order of 10s of µF (3.3 μF in [17,32] and 22 µF in [41]), which is very high compared to *C_P_*. Due to this *C_L_*, the PZT-generated voltage cannot exceed *V_RECT_* much, as shown in Figure 1b. This *C_L_* acts as protection against the strong input excitations, which can generate high piezoelectric open-circuit voltage (*V_OC_*). Thus, *V_RECT_* remains lower than the maximum voltage allowed by the process node (*V_MAX_*). However, as discussed earlier, the addition of a large load capacitor significantly reduces energy extraction from the PZT [28]. In contrast, SECE-based harvesting interfaces can suffer from strong input excitations with high *V_OC_*s of the PZT, where *V_P_* is proportional to the strength of the input excitation applied to the PZT. For weak input excitations, PZT-generated peak voltage remains lower than *V_MAX_*, but in the case of strong input excitations, *V_P_* can exceed *V_MAX_* (Figure 1d) and possibly damage the integrated circuit (IC). 

To address all of the abovementioned challenges, a novel harvesting interface circuit is proposed. Unlike prior works [12,29,37], this work proposes an integrated-circuit-based harvesting solution for multiple PZTs. The power stage is designed using bi-directional switches, which enables bi-directional energy transfer for both energy investment and storage, while only using a single inductor. The energy from a PZT is deposited to a temporary storage capacitor (*C_STOR_*) using an inductor at the peak voltage of *V_P_*. A control circuit monitors the PZT deformation and activates the harvesting interface to invest energy stored on *C_STOR_* back to the PZT (pre-biasing it) only when another deformation is detected. This energy-investment scheme is a new approach introduced in this work. Energy investment increases the PZT damping force, and more mechanical energy is converted to electrical energy. The PZT voltage keeps rising after this point until its peaking event is detected. In addition, to avoid IC damage due to strong excitations exceeding *V_MAX_*, another harvesting approach called partial electric charge extraction (PECE) [29] is adopted. In PECE, charges are partially extracted from the PZT whenever *V_P_* approaches *V_MAX_*, thereby allowing the harvesting interface circuit to handle strong input excitations without needing any load capacitance, thereby maximizing energy extraction. A dedicated control circuit is designed to control PECE cycles for multiple PZTs and their different polarities. In addition, energy investment and PECE in the same circuit with multiple PZTs require a special control circuit and can result in many different operation cases. For this purpose, special control circuits and sub-blocks were designed in this work. All these cases and operation phases make this work unique from previous works.

The rest of this paper is organized as follows. In Section 2, the key approaches of the proposed harvesting interface circuit are described. In Section 3, the proposed harvesting interface implementation details are elaborated. The simulation results and comparison with prior works are reported in Section 4, and the paper is concluded in Section 5.

## 2. Proposed Maximum Energy Extraction Approach

PZTs can be easily attached to human body joints to convert vibrations generated by human motion into electrical energy. To maximize energy extraction from human body motion, multiple PZTs can be attached to human body joints or skin. As human motion can be irregular, a PZT-harvesting interface circuit should be optimized to deal with these vibrations of random amplitude and frequency. Therefore, the proposed energy-harvesting interface is designed to harvest energy from irregular motion applied to multiple PZTs simultaneously. To maximize the extractable energy, an energy-investment scheme is introduced. In addition, to handle strong input excitations, which can generate *V_OC_*s greater than *V_MAX_*, the harvesting interface utilizes a PECE scheme [29]. 

### 2.1. Energy Investment

As explained earlier, biasing a PZT to a higher voltage level prior to bending deformation results in a higher amount of energy extraction from the PZT during deformation. Therefore, in the proposed energy-harvesting interface, an energy-investment scheme is introduced. In this scheme, energy is invested to a PZT at the start of its bending deformation, which increases the bias voltage across the PZT and makes its damping force stronger, resulting in higher energy extraction from the PZT. 

**Figure 2:**
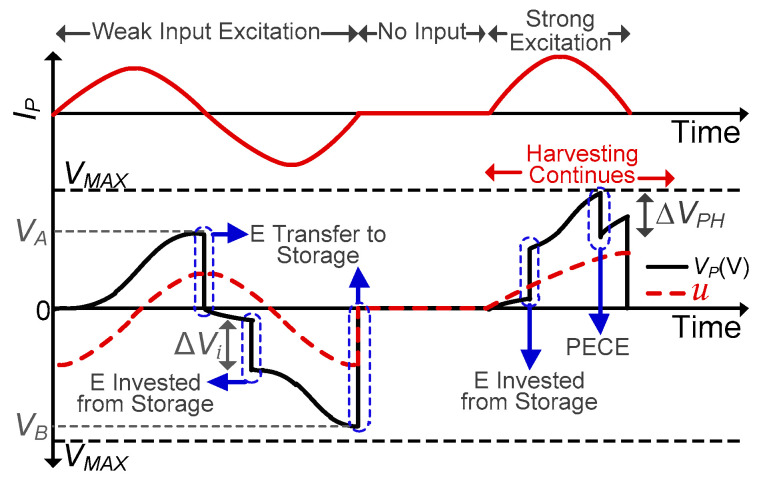
Conceptual waveform of proposed harvesting system with different input excitations.

The conceptual waveform of the proposed energy investment-based harvesting scheme is shown in Figure 2. A storage capacitor (*C_STOR_*) is utilized to temporarily store the PZT-generated energy and invest it back to the PZT when required. For weak input excitations (assuming *C_STOR_* is not fully charged initially), *V_P_* is maximized until it reaches its peak (*V_A_*), at which point some energy is transferred to *C_STOR_* through the inductor until it becomes fully charged and the remaining energy is harvested to the battery. The energy generated by the PZT till this point can be written as

(2) $$E_{A}=\;\frac{1}{2}C_{P}V_{A}^{2}$$

When the next deformation is detected, the energy from *C_STOR_* is invested to the PZT (using the inductor), which increases the PZT voltage by ∆*V_i_*, resulting in more energy extraction from the PZT until peak voltage (*V_B_*) is detected. The energy at this point (*V_B_*) can be written as

(3) $$E_{B}=\;\frac{1}{2}C_{P}V_{B}^{2}$$

Assuming that the same amount of deformation is applied to PZT in the second half cycle and the energy (∆*V_i_* = *V_A_* ideally) is invested as soon as PZT deformation starts, Equation (3) can be written as

(4) $$E_{B}=\;\frac{1}{2}C_{P}{\left(\Delta V_{i}+V_{A}\right)}^{2}=4\times E_{A}$$

*E_B_* is ideally the maximum harvestable energy, which is significantly higher than *E_A_*, thanks to energy investing. In a non-ideal situation, energy will be invested when *V_P_* has already increased to a threshold voltage (determined by the harvesting interface), as shown in the second half cycle in Figure 2. This means that the actual *E_B_* will not have reached a maximum amount as in (4), but still it would be significantly higher than *E_A_*. Therefore, in the proposed energy-investment scheme, the energy generated by the PZT is maximized by the energy investment to the PZT.

### 2.2. Partial Electric Charge Extraction (PECE)

PZT-generated energy is inversely proportional to the load capacitance seen by the PZT [28,29]. As the load capacitance is reduced, *E_PZT_* increases, but the resultant *V_P_* also increases. The IC could be damaged if this *V_P_* exceeds *V_MAX_*. At this point, the PZT needs to be discharged to protect the IC, but it is also desirable to maintain the PZT voltage as high as possible for maximum energy extraction, as explained earlier. Therefore, a PECE scheme is adopted in which once the PZT voltage approaches *V_MAX_*, charges are partially extracted from the PZT, thereby decreasing *V_P_* by PECE step voltage (∆*V_PH_*) after a single PECE cycle, as shown in Figure 2. Following a PECE cycle, the PZT voltage keeps increasing until another PECE condition (*V_P_* >= *V_MAX_*) is achieved or the peak voltage (*V_PK_*) is detected. Once *V_PK_* is detected, the charges are completely extracted from the PZT. By using this approach, energy extraction is maximized by load capacitance minimization while avoiding *V_MAX_*. This allows the IC to tolerate strong input excitations that can generate *V_OC_*s higher than *V_MAX_*.

Determining the optimal value of ∆*V_PH_* is critical for achieving maximum energy extraction from the PZT. Assuming a strong input excitation, if ∆*V_PH_* is too small, PECE will be activated many times before peak detection. This means that the harvesting interface will need to be activated multiple times to extract a partial amount of charges from the PZT, incurring switching losses and thereby reducing the net amount of harvested energy (*E_HRV_*). Whereas, if ∆*V_PH_* is too large, *V_P_* will drop to a low voltage, decreasing the damping force on the PZT and hence lowering the energy extraction from it. Therefore, determining the optimal value of ∆*V_PH_* is very important for maximum energy extraction from the PZT. A ∆*V_PH_* of 8 V was chosen based on simulations.

## 3. Multiple Piezoelectric Energy-Harvesting Interface Implementation

**Figure 3:**
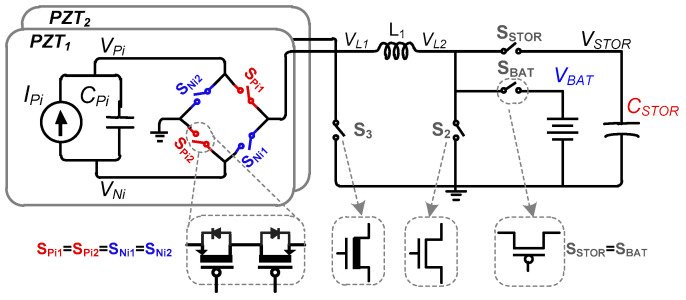
Proposed Dual-PZT energy-harvesting interface circuit.

To maximize the energy extraction from human body joints, multiple PZTs are utilized in this work. A simplified circuit diagram of the proposed piezoelectric energy-harvesting interface circuit is shown in Figure 3. For conceptual verification of high-voltage energy-harvesting using multiple PZTs, two PZTs are used in this work to harvest energy using a shared single inductor (*L_1_*). This inductor is used to transfer energy from the PZTs to the battery and/or storage capacitor (*C_STOR_*). Energy harvesting from *C_P_* to *C_STOR_* (or battery) and energy investment from *C_STOR_* to *C_P_* requires bidirectional power switches. Therefore, two switches per PZT, S_Pi1_ and S_Pi2_, are used for the positive half cycle of PZT-generated output voltage. Similarly, two switches per PZT, S_Ni1_ and S_Ni2_, are used for the negative half cycle. Altogether, these four switches act as a rectifier and are only activated according to the PZT-generated voltage polarity.

**Figure 4:**
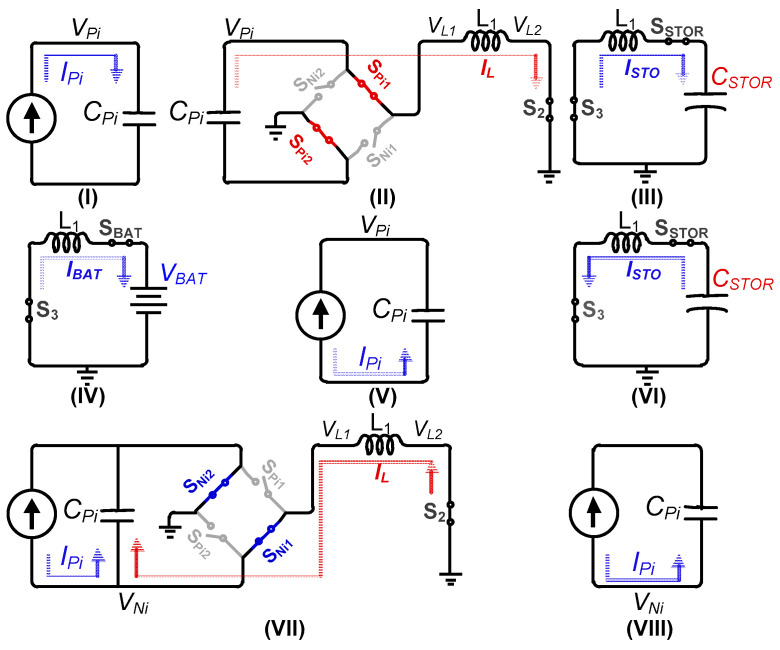
Operation phases of PZT energy-harvesting interface while activating only PZT_1_: (**I**) Positive *I_Pi_* charging *C_Pi_* until *V_Pi_* peaks, (**II**) *C_Pi_* drains into L_1_, (**III**) L_1_ transfers energy to *C_STOR_*, (**IV**) L_1_ drains into BAT, (**V**) Negative *I_Pi_* charging *C_Pi_* only, (**VI**) *C_STOR_* transfers energy to L_1_, (**VII**) L_1_ transfers energy to *C_Pi_* (energy investment), and (**VIII**) Negative *I_Pi_*, charging *C_Pi_* until *V_Ni_* peaks.

**Figure 5:**
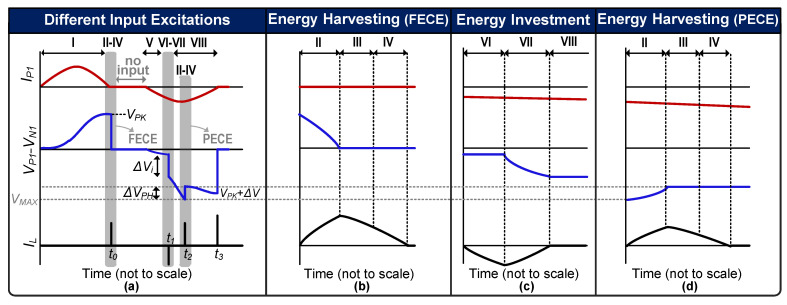
Conceptual waveforms of the proposed PZT energy-harvesting interface while activating only PZT_1_. (**a**) PZT-generated output for different positive and negative input excitations. (**b**) Zoomed waveform with FECE operation phases of energy storage and harvesting. (**c**) Zoomed waveform with operation phases of energy investment. (**d**) Zoomed waveform of a PECE cycle.

The proposed harvesting interface operation phases are shown in Figure 4, and the corresponding conceptual waveforms are shown in Figure 5. For convenience, in the harvesting operation explanation, only a single PZT (PZT_1_) is assumed to undergo deformation; therefore, the circuit configurations in Figure 4 and Figure 5 correspond to PZT_1_ only. Whenever mechanical deformation is applied to PZT_1_, the generated *I_P1_* starts charging *C_P_* (Phase I). *V_P1_* keeps rising until the peak voltage (*V_PK_*) is detected (at time t_0_), as shown in Figure 5a. At this point, S_P11_/S_P22_/S_2_ are turned on and the charges are completely extracted from *C_P_* to the inductor (*L_1_*) (Phase II). This will be referred to as full electric charge extraction (FECE) in the remainder of the paper. In Phase III, S_P11_/S_P22_/S_2_ are turned off and S_3_/S_STOR_ are turned on to transfer energy from *L_1_* to *C_STOR_* (Figure 5b) until *V_STOR_* reaches the desired maximum value (*V_STOR_*_(MAX)_). The remaining energy on the inductor (if any) is delivered to the battery in Phase IV by turning on S_3_/S_BAT_. Once this energy transfer is complete, S_3_/S_BAT_ are turned off to mark the completion of a harvesting cycle. 

Assuming there is no input excitation (between Phase IV and V in Figure 5a) applied to the PZT_1_ for a while, the circuit remains idle. Once another (second) deformation is applied to the PZT1 in the opposite direction, *I_P1_* starts charging *C_P1_* with opposite polarity (Phase V). As soon as this deformation is detected by the circuit, an energy investment cycle is initiated (at time t_1_ in Figure 5a). The zoomed energy investment phases VI–VII are shown in Figure 5c. In Phase VI, the energy stored on *C_STOR_* is transferred to the inductor by closing S_3_/S_STOR_. To invest this energy into *C_P1_*, the circuit is configured as shown in Phase VII in Figure 4, where the inductor charges *C_P1_* until *I_L_* becomes 0, which marks the end of the energy investment. During energy investment, *V_P1_* increases by ∆*V_i_*, as the waveform shows in Figure 5a. Due to this increased voltage, the damping force of the PZT increases, and more energy is extracted from PZT until *V_P1_* reaches the peak voltage (Phase VIII). After peak voltage detection (at time t_3_), similar steps (II–IV) as those performed for the 1st deformation for energy transfer to *C_STOR_* and battery are repeated. In the case of multiple PZTs, energy will be invested/harvested to/from whichever PZT deforms first or detects *V_PK_* first, while accounting for PZT voltage polarity. 

In some cases, after energy investment, *V_P_* can rise faster due to strong input excitations and can approach *V_MAX_* (as shown in the 2nd deformation in Figure 5a). In that case, PECE is activated (at time t_2_) and PZT voltage is decreased by ∆*V_PH_*, and thereby *V_P_* keeps increasing until *V_PK_*. PECE also repeats the same energy-harvesting steps (II–IV) as in the 1st deformation, as shown in Figure 5d. In another possible scenario (not shown in Figure 5) of strong input excitations where there is no energy available to invest at the start of the second deformation, the PZT voltage will keep rising until either the PECE condition is achieved or peak voltage is detected. For strong excitations, multiple PECE cycles can be activated before a peaking event is detected. 

**Figure 6:**
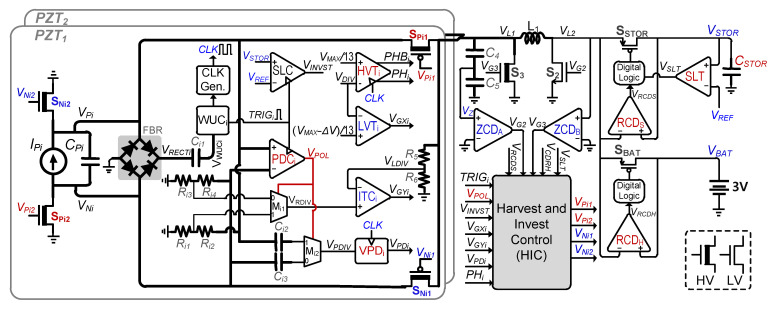
Top-level circuit of Dual-PZT energy-harvesting interface for energy investment and harvesting with time-shared inductor.

The top-level implementation details of the proposed piezoelectric energy-harvesting interface circuit are presented in Figure 6. A full-bridge rectifier (FBR) is used to rectify the PZT alternating output to *V_RECT_*. This rectifier is essential to interface the wakeup-controller (WUC) [37] with the PZT, which is used to monitor and detect PZT deformation. The WUC is implemented using all low-voltage (LV) devices except a high-voltage capacitor *C_i1_*. The *V_RECT_* is capacitively coupled using *C_i1_*, which is necessary to deal with the high output voltages of the PZT. This rectifier is not used for actual energy transfer to/from the PZT and is not required after PZT deformation has been detected. Once a deformation is applied to the PZT, the WUC monitors the rise in PZT-generated voltage. As *V_RECTi_* starts to increase, *V_WUCi_* starts to increase as well. The WUC triggers *TRIG_i_* signal only when *V_WUCi_* (*V_P_*) exceeds a certain threshold voltage level. After the trigger, one of the terminals in C_i1_ connected to WUC is grounded to avoid damaging WUC with increasing *V_P_* input. 

*TRIG_i_* is used to activate other sub-blocks using a specially designed harvest and invest control circuit (HIC). First of all, *TRIG_i_* activates the polarity detection controller (PDC), which determines the polarity of the PZT voltage by comparing *V_Pi_* and *V_Ni_*. Polarity is termed as positive (*V_POL_* = 1) for *V_Pi_* > *V_Ni_*; otherwise it is negative (*V_POL_* = 0). Along with the PDC, the WUC activates a storage level comparator (SLC), which is a clocked comparator, where clock in this case is a single pulse (*TRIG_i_*). The SLC is utilized to check the voltage level of the storage capacitor (*C_STOR_*). *V_STOR_* > *V_REF_* means the capacitor is charged with a certain voltage level and energy can be invested to the PZT, where *V_REF_* (internally generated reference voltage) in this case is 3.3 V. The SLC is time shared by both PZTs depending on whichever triggers first. In addition, *TRIG_i_* also activates a clock generator (CLK Gen.) [42] to generate a clock that is necessary for the operation of other sub-blocks. The activation and operation of other sub-blocks are explained in the following sub-sections.

### 3.1. Energy Investment Control

For maximum energy extraction from the PZT, energy investment to the PZT is carried out. Before energy investment, the SLC compares *V_STOR_* and *V_REF_* and determines if there is enough energy in *C_STOR_* available to invest. If *V_STOR_* > *V_REF_* condition is met, the energy investment cycle starts. In this cycle, energy is initially transferred from *C_STOR_* to *L_1_* by closing S_3_ and S_STOR_, as explained earlier in Figure 4. A zero-crossing detector (ZCD_B_) (derived from [43]) is activated to monitor this energy transfer. ZCD_B_ keeps comparing *V_L2_* with GND, and energy transfer to *L_1_* is stopped when *V_L2_* <= 0 V condition is met. At this point, energy can be invested from the inductor to the PZT by turning on power switches according to the polarity of the PZT. For positive polarity, S_Pi1_/S_Pi2_ are closed along with S_2_, and energy starts transferring from *L_1_* to the PZT. Here, *V_L1_* starts rising and so does *V_Pi_*. As both of these voltages can be higher than 5 V, they cannot be handled with LV switches. To handle these higher voltages, *V_L1_* is divided by *R_5_* and *R_6_* to *V_LDIV_*, and *V_Pi_* is divided by *R_i1_* and *R_i2_*, and then this divided voltage is fed to a 2 × 1 analog multiplexer (M_i1_) whose output (*V_RDIV_*) is selected by *V_POL_*. A comparison of *V_RDIV_* with *V_LDIV_* is performed by an investment-tracking comparator (ITC), which keeps tracking these voltages until *V_LDIV_* becomes lower than *V_RDIV_*, which means *V_L1_* < *V_Pi_* and *I_L_* becomes ~0 A. At this point, the investment cycle is completed and S_Pi1_/S_Pi2_/S_2_ are opened. *I_Pi_* keeps charging *C_Pi_* after this point until the end of deformation.

### 3.2. Peak Voltage Detection Control

Once the investment has been completed, the PZT voltage must increase to its peak to maximize energy extraction. *V_PK_* can be detected by a clocked voltage peak detector (VPD_i_) (based on [37]), which is activated by the PDC. Each PVD (just like WUC) is implemented using all LV devices except a high voltage capacitor. The PZT voltage is capacitively coupled using this capacitor, which is necessary to handle the high output voltages of the PZT. Therefore, two capacitors, *C_i2_* and *C_i3_*, are utilized to deal with the positive and negative polarity of the PZT voltage, respectively. *V_POL_* determines the output (*V_PDIV_*) of another 2 × 1 analog Multiplexer (M_i2_) for VPD_i_. *V_PDIV_* is injected to VPD_i_, which keeps tracking the PZT voltage for *V_PK_*. VPD_i_ determines the *V_PK_* by comparing the slope of PZT voltage during two consecutive clock cycles. At the end of a single deformation, *I_Pi_* becomes ~0 A and PZT voltage slope becomes negative, which signals peak detection. Voltage at the output of the capacitor (either *C_i2_* / *C_i3_*) is repeatedly reset to ground at the end of every clock cycle to keep their voltage within the range of 0 V-V_DD_, preventing damages on IC due to high voltages. The HIC also keeps monitoring the output of the VPDs of both PZTs in case both of them are pressed together. If the *V_PK_*s of both PZTs are detected at the same time, the first PZT to transfer energy to storage/battery through the inductor is selected by a priority bit. Following the energy transfer from the first PZT, energy is extracted from the second PZT immediately. To avoid false peak detection, the HIC stops peak voltage detection during the energy investment/harvesting cycles of either PZT. This does not affect the overall performance of the system as energy investment/harvesting cycles are completed within a few micro-seconds.

### 3.3. PECE Control

Partial electric charge extraction (PECE) control has also been adopted to extract more energy from the PZT for *V_OC_*s greater than the maximum tolerable voltage (*V_MAX_*). The supply voltage (*V_DD_*) used for control circuits in this work is 3.3 V. The transistors used for control circuits can only handle up to 5 V. Along with VPD, a clocked high-voltage tracker (HVT) is also activated to track *V_Pi_* for *V_MAX_*. A resistive divider is utilized to generate fractional PZT voltage (*V_DIV_*) with a division ratio of 13:1, which is necessary to keep divided *V_RECT_* (*V_DIV_*) within the range of *V_DD_*. Here, *V_DIV_* is also generated through resistive dividers just like *R_1i_*-*R_4i_*, followed by a *V_POL_*-controlled (2 × 1) analog multiplexer, which are not shown here to avoid figure complexity. The HVT keeps comparing *V_DIV_* with *V_MAX_* /13 until the *V_DIV_* >= *V_MAX_* /13 condition is met, which initiates a PECE cycle. During PECE, the PZT voltage is decreased by PECE step voltage (∆*V_PH_*) during energy transfer to *L_1_*. A clocked low-voltage tracker (LVT) is activated to keep monitoring the PZT voltage and detect the point where *V_DIV_* becomes lower than ((*V_MAX_* − ∆*V*)/13). At this point, energy transfer to the inductor is stopped and PZT voltage keeps rising until another PECE condition is met or *V_PK_* is detected.

### 3.4. Harvesting and Storage Control

Once a PECE condition is met or peak voltage is detected, all of the energy from the PZT is transferred to the inductor. A zero-crossing detector (ZCD_A_) is utilized to monitor this energy transfer. A capacitive divider (*C_4_*, *C_5_*) is necessary here to generate a low voltage (*V_Z_*) from high voltage (*V_L1_*) as ZCD_A_ consists of LV transistors. The ZCD keeps comparing this *V_Z_* with GND until the *V_Z_* <= 0 V condition is achieved, which indicates peak inductor current (*I_PK_*). After this, the HIC determines whether energy will be transferred to *C_STOR_* and/or the battery. As soon as energy transfer to the inductor is started, the HIC starts evaluating *C_STOR_* with the SLC. The harvesting and storage decisions made by the HIC based on this evaluation are explained with the following 2 cases, and the conceptual waveforms are shown in Supplementary Figure S1.

#### 3.4.1. Case1: *C_STOR_* Not Fully Charged

In case ①, where *C_STOR_* is not fully charged (*V_STOR_* < *V_REF_*), energy is transferred from the inductor to *C_STOR_*. Here, a storage-level tracker (SLT) is activated to keep monitoring *V_STOR_* until *V_STOR_* >= *V_REF_* condition is achieved. At this point, the remaining energy (if any) on the inductor is transferred to the battery. Energy transfer to the battery is monitored by a reverse current detector (RCD_H_), which blocks reverse current and detects the point where *I_L_* becomes ~0 A, marking the completion of a harvesting cycle. However, if the energy on the inductor is not enough to fully charge *C_STOR_*, the *V_STOR_* >= *V_REF_* condition will never be achieved, and reverse energy transfer from *C_STOR_* to *L_1_* can happen, which can waste energy. Therefore, to prevent this situation, another reverse current detector (RCD_S_) is utilized. HIC activates RCD_S_ along with SLT, where RCD_S_ continues monitoring the energy transfer from the inductor to *C_STOR_* and detects the point where *V_L2_* becomes lower than *V_STOR_*, which means that *I_L_* is ~0 A. Here the energy transfer to *C_STOR_* is stopped and a single harvesting cycle (or PECE cycle) is completed. 

If the *V_STOR_* >= *V_REF_* condition is detected by the SLT while *I_L_* is non-zero, this means that there is still some energy on the inductor, which can be transferred to the battery. However, in some cases, this energy can be very low, and as soon as S_BAT_ is closed, the circuit will only see reverse current, which makes RCD_H_ detection impossible and the circuit fails. Therefore, *V_BAT_* (3 V) is intentionally kept lower than *V_STOR_* (3.3 V) to avoid circuit failure in the case of lower energy on the inductor during case ①. In this case, RCD_H_ is activated together with the SLT and RCD_S_. During energy transfer from *L_1_* to *C_STOR_*, *V_L2_* starts rising slowly until it becomes >3 V. RCD_H_ detects this point, and its output (*V_RCDH_*) becomes low, which means even if there will be little or no energy available on the inductor, once the energy transfer to *C_STOR_* is complete, RCD_H_ will still detect *I_L_* becoming ~0 A (by detecting *V_RCDH_* high at *V_L2_* < *V_BAT_*). This will de-activate RCD_H_ and mark the completion of a harvesting cycle.

#### 3.4.2. Case2: *C_STOR_* Fully Charged

In case ②, where *C_STOR_* is already fully charged, the *V_STOR_* >= *V_REF_* condition is detected by the HIC. Therefore, in this case, all of the energy on the inductor needs to be transferred to the battery. Here only RCD_H_ is activated to block reverse current by detecting the point where *I_L_* becomes ~0 A.

The HIC plays a vital role in determining energy transfer cycles. In another case, where both PZTs are pressed together, energy is only invested to the one whose WUC triggers first, and only the VPD of the second PZT is activated. The inductor is time shared between both PZTs for energy transfer in case both PZTs detect *V_PK_* or meet the PECE condition at the same time. Similarly, the inductor is also time-shared in case the PECE condition is achieved by one PZT and voltage peak is detected by the other PZT. This time sharing is possible because energy transfer to/from the inductor to/from the PZT only takes a few µs. This makes the circuit reliable for irregular human motion conditions. To deal with the high voltage of PZTs, S_Pi1_/S_Pi2_/S_Ni1_/S_Ni2_/S_3_ are designed using high-voltage (HV) transistors, whereas S_2_/S_BAT_/S_STOR_ are designed using LV transistors to minimize conduction loss.

### 3.5. Bi-Directional Control

**Figure 7:**
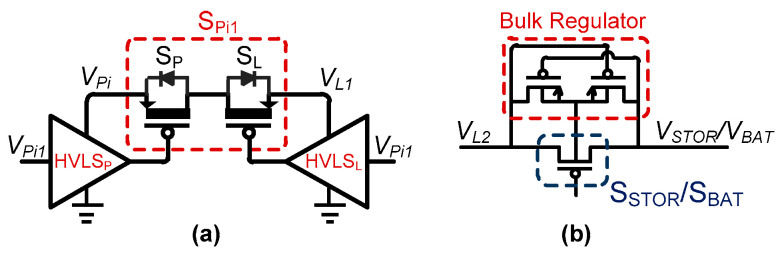
(**a**) Bi-directional switch(s) between PZTs and Inductor (S_Pi1_, S_Ni1_). (**b**) Bulk Regulator for S_STOR_ and S_BAT_.

**Figure 8:**
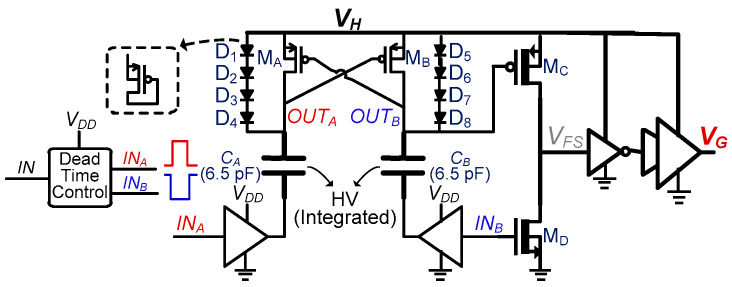
High-voltage level shifter (HVLS) for bi-directional switches between PZTs and inductor.

For the proposed dual-PZT harvesting circuit, S_Pi1_/S_Pi2_/S_Ni1_/S_Ni2_ need to be bi-directional for energy investment and harvesting. Therefore, bi-directional transistors, as shown in Figure 7a, were utilized for these switches. Depending on energy investment to the PZT_i_ or energy extraction from the PZT_i_, the voltage on either side of the switch can be higher. This means that the body of these switches cannot be tied to either side. Therefore, each switch consists of two transistors, S_P_ (PZT side) and S_L_ (inductor side). A carefully designed high-voltage level shifter (HVLS) is used to control these switches, as shown in Figure 8. These two switches (with HVLS) are necessary for (1) blocking current from PZT_i_ to the inductor when *V_Pi_* is increasing, (2) blocking current from PZT_1_ to/from PZT_2_, and (3) blocking current to PZT_1_ during energy investment to PZT_2_ and vice versa. The *V_H_* node in the HVLS is interfaced with the PZT or the inductor to generate a gate control voltage (*V_G_*) to turn on/off these power switches with full voltage swing. *V_Pi1_* is the trigger signal (for HVLS) to turn on/off S_Pi1_. Similarly, *V_Pi2_*, *V_Ni1_*, and *V_Ni2_* (shown in Figure 6) are trigger signals used for S_Pi2_, S_Ni1_, and S_Ni2_, respectively. All these signals are activated by HIC for harvesting/storage/invest operation.

S_STOR_ is a low-voltage switch that needs to be bi-directional for energy transfer to/from the inductor. Voltage on either side of S_STOR_ (and S_BAT_) can be high; therefore, the body of these switches also needs to be attached to the high-voltage side. In this case, a bulk regulator [27] (Figure 7b) is utilized to connect the high voltage to the body of the switch to block the reverse current. 

As stated earlier, HVLS is needed to generate a gate control voltage with full voltage swing (up to 40 V). Whereas, supply voltage used to control HVLS is <5 V, which is necessary to minimize switching losses. The low-voltage input signals cannot be directly applied to the high-voltage transistors of HVLS, which are at a different potential. That is why a capacitively cross-coupled HVLS is utilized. M_B_ is intentionally kept larger than M_A_. Initially, IN_B_ is kept high. As *V_H_* starts to increase, OUT_B_ becomes high, which keeps M_A_ remained closed. Diodes D_1_-D_4_ are used to charge *C_A_* to maintain OUT_A_ high enough to keep voltage across M_A_ < 5 V, as M_A_ and M_B_ are made with LV devices. Similarly, D_5_-D_8_ are utilized for C_B_ and M_B_. As OUT_B_ follows *V_H_*, M_C_ remains closed and *V_FS_* remains low, *V_G_* keeps following *V_H_*. To pull down *V_G_*, IN_B_ is turned low, which pulls down OUT_B_ and enough gate-source voltage difference is created to turn on M_C_. *V_FS_* quickly rises to the (high voltage) level of *V_H_*, which triggers inverters to invert *V_G_* value as well. Inverters/buffers on the right side of Figure 8 are made with HV transistors; therefore, they can handle full voltage swing.

The main target of this work was to perform efficient harvesting operation while assuming external power supply is available. Therefore, this work mainly focused on high-voltage energy harvesting challenges using multiple PZTs and cold-start circuit implementation is not considered.

## 4. Results and Discussion

**Figure 9:**
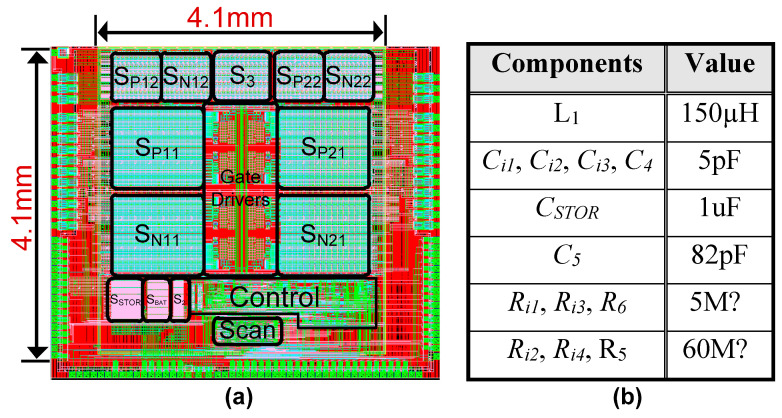
(**a**) Chip layout. (**b**) Components used for harvester simulations.

**Figure 10:**
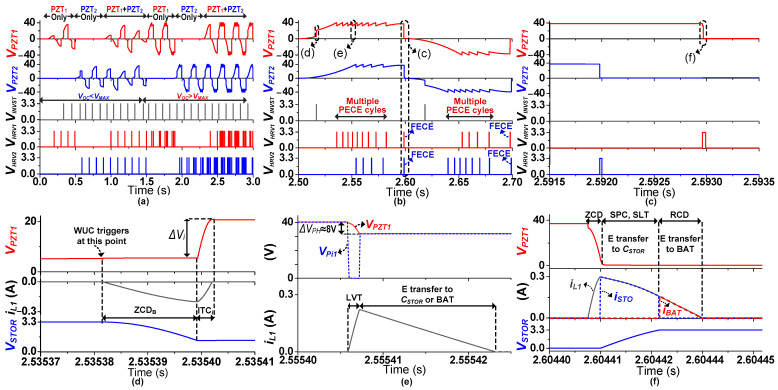
Simulation results of dual-PZT energy-harvesting circuit. (**a**) Random input excitations applied to both PZTs separately and simultaneously. (**b**) Zoomed output voltages of both PZTs increasing simultaneously. (**c**) Harvesting signal sequences after peak detection. (**d**) An energy-investment cycle of PZT_1_. (**e**) A PECE cycle of PZT_1_. (**f**) An FECE cycle after peak detection.

The proposed energy-harvesting interface was designed and simulated in 350 nm BCD process. Figure 9a shows the layout of the proposed harvesting interface, which occupies an active area of 16.81 mm^2^. Figure 9b shows the values of components used for proposed harvesting interface. For simulations, a PZT electrical model with 20 nF internal capacitance (*C_P_*) was utilized. Figure 10 shows the simulation results of the proposed energy-harvesting interface with different input excitations to demonstrate the reliability of the harvesting controller (HIC). Initially, only PZT_1_ is pressed (assumed); therefore, *V_PZT1_* (PZT_1_-generated voltage) increases and energy is harvested at every peak. The harvesting cycle activation for PZT_1_ is represented by the *V_HRV1_* signal in Figure 10a. During weaker input excitations, *V_PZT1_* remains lower than *V_MAX_*, and the charges are completely extracted at the peak voltage. Similarly, weak input excitations are applied to only PZT_2_, which triggers *V_HRV2_* for harvesting at the peak of *V_PZT2_* (PZT_1_-generated voltage). After that, weak input excitations are applied to both PZTs simultaneously to verify the effectiveness of the HIC to handle both PZTs at the same time. *V_INVST_* here refers to the activation of an energy investment cycle, where energy is invested to only one of the PZTs if both PZTs are pressed together. 

As stronger input excitations are applied to PZT_1_, *V_PZT1_* can exceed *V_MAX_*. Therefore, PECE is initiated to keep *V_PZT1_* below *V_MAX_* and maximize energy extraction from PZT_1_. *V_HRV1_* here represents the activation of a PECE cycle as well, which is basically a partial harvesting cycle. Therefore, *V_HRV1_* can be seen as activated multiple times in Figure 10a as PECE is initiated a few times. Similar steps are repeated for PZT_2_ with stronger input excitations. In the end, both PZTs are pressed simultaneously with stronger input excitations. It can be concluded from Figure 10a that the proposed HIC effectively differentiates between different voltage polarities of both PZTs to initiate energy investment or harvesting cycles accordingly.

Figure 10b shows a zoomed waveform of the case where both PZTs are pressed simultaneously. Initially, (for positive polarity) *V_PZT1_* rises faster than *V_PZT2_*; therefore, energy is invested to PZT_1_, and *V_PZT1_* keeps rising until the *V_OC_* >= *V_MAX_* condition is met. At this point, charges are partially extracted (PECE) from PZT_1_. Multiple PECE cycles are activated (represented by *V_HRV1_*) until the peak detection of *V_PZT1_*. *V_PZT2_* also keeps rising without energy investment with a few PECE cycles (represented by *V_HRV2_*) until its peak voltage is detected. 

Once the peak voltage is detected, charges are completely extracted from both PZTs. The similar operation of energy harvesting is shown for both PZTs when their output voltages have negative polarity. PECE as well as FECE takes a very short time (a few μs) for completion. A zoomed version of FECE activation for both PZTs sharing a single inductor is shown in Figure 10c. In the given case, the *V_PZT2_* peak is detected earlier than the *V_PZT1_* peak, and therefore, *V_HRV2_* triggers to perform FECE on PZT_2_. Following *V_PZT1_* peak detection, *V_HRV1_* is activated to perform FECE on PZT_1_, as well. 

The zoomed waveform of an energy investment cycle of PZT_1_ is shown in Figure 10d. As the WUC triggers *TRIG_1_*, the HIC starts the evaluation of *C_STOR_* using the SLC, which triggers *V_INVST_* only after assuring that *C_STOR_* is fully charged. Energy is extracted from *C_STOR_* using the inductor, where ZCD_B_ monitors this energy transfer to stop it once *V_L2_* <= 0 V is detected. Later, this energy on the inductor is transferred (invested) to PZT_1_, where *V_PZT1_* can be seen rising by ∆*V_i_*) in Figure 10d at the end of the transfer. Whereas, for PZT_2_ (in Figure 10b), after the WUC triggers *TRIG_2_*, the SLC does not trigger *V_INVST_* because the energy from *C_STOR_* has already been invested to PZT_1_. In that case, VPD_2_ immediately starts tracking the peak of *V_PZT2_*. 

After energy investment to PZT_1_, VPD_1_ also keeps tracking the peak of *V_PZT1_*. At the same time, HVT_1_ of PZT_1_ and HVT_2_ of PZT_2_ are also active to track the respective PZT’s voltage for *V_MAX_*. Due to stronger input excitation applied to PZT_1_, HVT_1_ detects the *V_PZT1_* > *V_MAX_* condition earlier, and PECE cycles are activated multiple times before the PZT_1_ peak voltage is detected by VPD_1_. During a PECE cycle, charges are partially extracted from a PZT using the inductor, as shown in Figure 10e. In PECE, energy is transferred from a PZT to the inductor for a short duration, during which *V_PZT1_* decreases by PECE step voltage (∆*V_PH_*). The LVT keeps monitoring *V_PZTi_* to stop this energy transfer once *V_PZTi_* decreases by ∆*V_PH_*. This energy on the inductor is later transferred to the storage, which marks the end of a single PECE cycle. 

Once either a PECE activation condition or peaking event is detected, energy needs to be harvested to *C_STOR_* and/or the battery, as explained earlier in Section 3.4. Figure 10f shows a case where energy is stored to both *C_STOR_* and the battery since the *C_STOR_* is not fully charged. After peak detection, the charges are completely extracted from PZT_1_ using the inductor (*V_PZT1_* becomes ~0 V at the peak of *i_L1_*). After that, energy is harvested to the *C_STOR_* first, until the *V_STOR_* >= *V_REF_* condition is met. The remaining energy on the inductor is transferred to the battery. The inductor current during energy transfer to *C_STOR_* (*i_STO_*) is denoted by the dashed blue line, whereas, the inductor current during energy transfer to the battery (*i_BAT_*) is denoted by the dashed red line in Figure 10f. 

**Figure 11:**
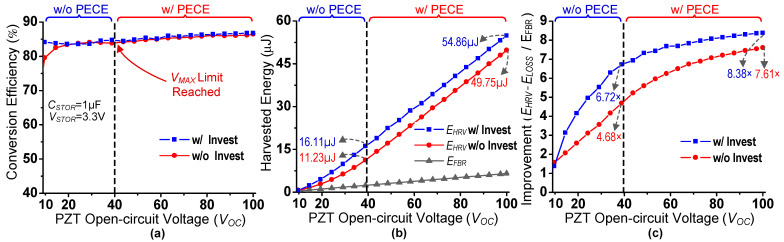
Simulation results of dual-PZT energy-harvesting circuit for different *V_OC_*s. (**a**) Harvesting circuit conversion efficiency. (**b**) Proposed circuit harvested energy (w/ and w/o energy investment) comparison with FBR-based harvester. (**c**) Energy extraction improvement compared to FBR-based harvester.

The performance and effectiveness evaluation of the proposed piezoelectric energy-harvesting interface is carried out with simulations with varying strengths of input excitations. Figure 11 shows the simulation results of the proposed harvesting interface for quantitative analysis. Unlike most of the prior works, this work deals with the discontinuous input pulses; therefore, quantitative analysis could be carried out in terms of energy, as presented in some of the recent works [28,29,37,44]. The energy harvested using the proposed harvesting interface in a single deformation cycle is represented as *E_HRV_*, and the energy consumed during this single harvesting operation is termed *E_LOSS_*. The *E_HRV_* can be calculated with the following equation:

(5) $$E_{HRV}=\;V_{BAT}\int_{to}^{tf}I_{BAT}\;dt$$

where *i_BAT_* is current transferred to the battery. The *V_BAT_* is assumed fixed and the *i_BAT_* is integrated for the duration (time t_o_ to t_f_) of energy transfer from inductor to the battery. For indirect comparison with state-of-the-art works, an FBR-based harvester is utilized. Energy harvested using this FBR-based harvester is indicated as *E_FBR_*. The results were recorded for harvesting operation with energy investment as well as without energy investment. The proposed harvesting interface maintains a good end-to-end conversion efficiency of >80% for almost all input excitation values, as shown in Figure 11a. The proposed harvesting interface harvests the PZT energy with PECE for input excitations when *V_OC_* >= 40 V; otherwise PECE is not used. With very weak input excitations (*V_OC_* < 15 V), without any energy investment, the conversion efficiency remains lower than when energy is invested. This is because the overall generated and transferred energy values are lower (without energy investment), and hence losses dominate. 

*E_HRV_* is compared with *E_FBR_* in Figure 11b. *E_HRV_* remains well above *E_FBR_* for different values of *V_OC_*s for harvesting with or without energy investment. To verify the effectiveness of the proposed harvesting circuit with strong excitations, PECE is performed with a *V_OC_* of 100 V. The difference between *E_HRV_* with and without energy investment can be seen as increasing up till 40 V, which verifies effectiveness of energy investment. However, this difference remains almost the same once *V_MAX_* is reached, as from this point, the energy extracted during each PECE cycle remains almost same. 

Energy extraction improvement using the proposed harvesting interface is compared with an FBR-based harvester in Figure 11c. Without energy investment (PECE for *V_OC_* > 40 V), the proposed circuit achieves up to 7.61× energy extraction improvement compared to an FBR-based harvester. In contrast, operating with both energy investment and PECE results in an energy extraction improvement of up to 8.38×. As *V_OC_* increases, the difference between improvement with and without energy investment decreases as energy extracted by PECE cycles starts to dominate. 

Table 1 summarizes the comparison between the proposed harvesting interface and state-of-the-art harvesting circuits [13,17,25,33,35,36]. To handle irregular input excitations, instead of flipping *C_P_* polarity in every half cycle to extract more energy in the next cycle [32] or using no flipping at all in [33], the proposed circuit temporarily stores energy on a storage capacitor and waits for the next vibration cycle to invest the stored energy to the PZT. In addition, prior works lack any kind of circuit in case the PZT-generated voltage exceeds the maximum voltage tolerated by the technology used. Therefore, PECE is utilized in this work for overvoltage protection as well as to ensure maximum energy extraction by keeping the PZT voltage high. With the help of PECE, the proposed harvesting interface can harvest from theoretically unlimited open-circuit voltages generated by the PZT, assuming that the input current remains within the manageable range. PECE performance is also limited by clock frequency used for tracking PZT voltage for *V_MAX_*. In addition, energy transfer time (from PZT to the inductor and then inductor to the battery) is also an important factor to determine maximum voltage limit, as there can be only so many energy-transfer cycles (of few µs) during a single deformation. With the help of energy investment and PECE, the proposed harvesting interface successfully harvests energy from excitations with *V_OC_* up to 100 V and achieves 8.38× energy extraction improvement compared to an FBR-based harvesting circuit. 

## 5. Conclusions

A novel dual piezoelectric energy investment and harvesting scheme is introduced in this work. An efficient harvesting controller is designed to time-share a single inductor for both energy extraction and investment to/from both PZTs. A WUC is utilized to monitor the PZT-generated output voltage and detect and trigger once this voltage approaches a certain threshold level. The WUC triggers the harvesting controller to start energy investment to the PZT using a temporary storage capacitor. Following energy investment, the PZT voltage continues to rise until a PECE condition (the PZT voltage exceeds the maximum tolerable voltage) is achieved or peak voltage is detected. In both conditions, energy is transferred to the inductor, keeping account of the correct polarity of the PZT-generated voltage. The proposed harvesting interface circuit was designed in 350 nm process. Without energy investment (PECE for *V_OC_* > 40 V), the proposed circuit achieves up to 7.61× energy extraction improvement compared to an FBR-based harvester. In contrast, operating with both energy investment and PECE results in an energy extraction improvement of up to 8.38×. With the help of energy investment and PECE, the proposed harvesting interface successfully harvests energy from excitations with *V_OC_* up to 100 V.

## Figures and Tables

**Table 1:** Comparison with prior arts.

	This Work	[17] ESSCIRCʹ 18	[25] JSSCʹ 10	[25] ISSCCʹ 19	[33] ISSCCʹ 18	[36] ISSCCʹ 18	[13] VLSIʹ 15
**Process**	350 nm BCD (40 V)	130 nm CMOS (Standard Voltage)	350 nm (Standard Voltage)	350 nm (Standard Voltage)	40 nm CMOS (10 V)	350 nm (Standard Voltage)	250 nm CMOS (40 V)
**Active Area (mm^2^)**	4.1 × 4.1	0.53	4.25	1.4 × 2.9	1.1 × 0.5	0.27	2 × 1.2
**Harvesting Technique**	Energy Invest + PECE	SSHI	SSHI	Reconfigurable VM-SECE	SECE	Delayed-SECE	Series-Parallel SC
**Harvesting Source**	PZT	PPA1022	MIDE V22B	Custom PZT/Nickel/PZT	MIDE PPA1011	MIDE V21B	PMN-PT On disc
**Source Capacitance (*C*_P_)**	20 nF (each PZT)	14 nF	12 nF	17 nF–49 nF	43 nF	-	150 nF
**Inductor**	220 μH	47 μH	0.82 mH	2.2 mH	2.2 mH	-	470 μH
**Excitation Type**	Irregular Pulse	Periodic	Periodic	Periodic & Shock	Periodic & Shock	Periodic	Irregular Pulse
**Input Voltage**	>100 V * (Theoretically unlimited)	2.5 V	2.4 V	<5 V **	<6 V **	<4 V **	35 V
Max. Improvement ***	7.61× (PECE only) 8.38× (PECE+ Invest)	3.85×	4×	5.11×	4.2×	-	-

* Open-circuit voltage. ** Estimated from paper. *** (*E_HRV_* − *E_LOSS_*)/*E_FBR_*.

## Data Availability

The data presented in this study are available within the article and supplementary material.

## Supplementary Materials

The following are available online at https://www.mdpi.com/article/10.3390/s21072357/s1, Figure S1: Conceptual waveform for the cases of harvesting and storage decisions made by HIC (a) Energy transferred to both *C*_STOR_ and battery, where SLT determines the end of energy transfer to *C*_STOR_ (b) Energy transferred to battery only (c) Energy transferred to *C*_STOR_ only, where RCD_S_ determines the end of energy transfer to *C*_STOR_ (d) Energy transferred to both *C*_STOR_ and battery, where SLT determines the end of energy transfer to *C*_STOR_ and RCD_H_ immediately determines end of energy transfer to the battery.

## References

[1] Li J., Dong Y., Park J.H., Lin L., Tang T., Zhang M., Wu H., Zhang L., Tan J.S.Y., Yoo J. Human-Body-Coupled Power-Delivery and Ambient-Energy-Harvesting ICs for a Full-Body-Area Power Sustainability. Proceedings of the 2020 IEEE International Solid-State Circuits Conference-(ISSCC).

[2] Kim J.-T., Heo B.-R., Kwon I. (2021). An Energy-Efficient UWB Transmitter with Wireless Injection Locking for RF Energy-Harvesting Sensors. Sensors.

[3] Khan D., Abbasizadeh H., Kim S.-Y., Khan Z., Shah S., Pu Y., Hwang K., Yang Y., Lee M., Lee K.-Y. (2018). A Design of Ambient RF Energy Harvester with Sensitivity of −21 dBm and Power Efficiency of a 39.3% Using Internal Threshold Voltage Compensation. Energies.

[4] Nguyen C.V., Nguyen M.T., Quyen T.V., Le A.M., Masaracchia A., Nguyen H.T., Nguyen H.P., Nguyen L.D., Nguyen H.T., Nguyen V.Q. (2020). Hybrid Solar-RF Energy Harvesting Systems for Electric Operated Wheelchairs. Electronics.

[5] Yu G., Chew K.W.R., Sun Z.C., Tang H., Siek L. (2015). A 400 nW Single-Inductor Dual-Input–Tri-Output DC–DC Buck–Boost Converter With Maximum Power Point Tracking for Indoor Photovoltaic Energy Harvesting. IEEE J. Solid State Circuits.

[6] Goeppert J., Manoli Y. (2016). Fully Integrated Startup at 70 mV of Boost Converters for Thermoelectric Energy Harvesting. IEEE J. Solid State Circuits.

[7] Peng Y., Choo D.K., Oh S., Lee I., Jang T., Kim Y., Lim J., Blaauw D., Sylvester D. An Adiabatic Sense and Set Rectifier for Improved Maximum-Power-Point Tracking in Piezoelectric Harvesting with 541% Energy Extraction Gain. Proceedings of the Digest of Technical Papers-IEEE International Solid-State Circuits Conference.

[8] Ye J., Tanzawa T. (2020). An Optimum Design of Clocked AC-DC Charge Pump Circuits for Vibration Energy Harvesting. Electronics.

[9] Teso-Fz-Betoño D., Aramendia I., Martinez-Rico J., Fernandez-Gamiz U., Zulueta E. (2020). Piezoelectric Energy Harvesting Controlled with an IGBT H-Bridge and Bidirectional Buck–Boost for Low-Cost 4G Devices. Sensors.

[10] Leicht J., Manoli Y. (2017). A 2.6 µW –1.2 mW Autonomous Electromagnetic Vibration Energy Harvester Interface IC with Conduction-Angle-Controlled MPPT and up to 95% Efficiency. IEEE J. Solid State Circuits.

[11] Park I., Maeng J., Lim D., Shim M., Jeong J., Kim C. A 4.5-to-16μW integrated triboelectric energy-harvesting system based on high-voltage dual-input buck converter with MPPT and 70V maximum input voltage. Proceedings of the Digest of Technical Papers-IEEE International Solid-State Circuits Conference.

[12] Khan M.B., Kim D.H., Han J.H., Saif H., Lee H., Lee Y., Kim M., Jang E., Joe D.J., Lee K.J. (2020). A Harvesting Circuit for Flexible Thin Film Piezoelectric Generator Achieving 562% Energy Extraction Improvement with Load Screening. IEEE Trans. Ind. Electron..

[13] Yang J., Lee M., Park M.-J., Jung S.-Y., Kim J. A 2.5-V, 160-μJ-output piezoelectric energy harvester and power management IC for batteryless wireless switch (BWS) applications. Proceedings of the 2015 Symposium on VLSI Circuits (VLSI Circuits).

[14] Beeby S.P., Tudor M.J., White N.M. (2006). Energy harvesting vibration sources for microsystems applications. Meas. Sci. Technol..

[15] Mitcheson P.D., Yeatman E.M., Rao G.K., Holmes A.S., Green T.C. (2008). Energy harvesting from human and machine motion for wireless electronic devices. Proc. IEEE.

[16] Tang G., Yang B., Liu J., Xu B., Zhu H., Yang C. (2014). Development of high performance piezoelectric d33 mode MEMS vibration energy harvester based on PMN-PT single crystal thick film. Sens. Actuators Phys..

[17] Javvaji S., Singhal V., Menezes V., Chauhan R., Pavan S. Multi-Step Bias-Flip Rectification for Piezoelectric Energy Harvesting. Proceedings of the ESSCIRC 2018-IEEE 44th European Solid State Circuits Conference (ESSCIRC).

[18] Mystkowski A., Ostasevicius V. (2020). Experimental study of macro fiber composite-magnet energy harvester for self-powered active magnetic bearing rotor vibration sensor. Energies.

[19] Lin S.C., Lee B.S., Wu W.J., Lee C.K. Multi-cantilever piezoelectric MEMS generator in energy harvesting. Proceedings of the Proceedings-IEEE Ultrasonics Symposium.

[20] Al-Ashtari W., Hunstig M., Hemsel T., Sextro W. (2013). Enhanced energy harvesting using multiple piezoelectric elements: Theory and experiments. Sens. Actuators Phys..

[21] Erturk A., Inman D.J. (2008). A distributed parameter electromechanical model for cantilevered piezoelectric energy harvesters. J. Vib. Acoust. Trans. ASME.

[22] DuToit N.E., Wardle B.L. (2007). Experimental verification of models for microfabricated piezoelectric vibration energy harvesters. AIAA J..

[23] Guyomar D., Badel A., Lefeuvre E., Richard C. (2005). Toward energy harvesting using active materials and conversion improvement by nonlinear processing. IEEE Trans. Ultrason. Ferroelectr. Freq. Control.

[24] Roundy S., Wright P.K. (2004). A piezoelectric vibration based generator for wireless electronics. Smart Mater. Struct..

[25] Ramadass Y.K., Chandrakasan A.P. (2010). An Efficient Piezoelectric Energy Harvesting Interface Circuit Using a Bias-Flip Rectifier and Shared Inductor. IEEE J. Solid State Circuits.

[26] Ottman G.K., Hofmann H.F., Bhatt A.C., Lesieutre G.A. (2002). Adaptive piezoelectric energy harvesting circuit for wireless remote power supply. IEEE Trans. Power Electron..

[27] Hehn T., Hagedorn F., Maurath D., Marinkovic D., Kuehne I., Frey A., Manoli Y. (2012). A fully autonomous integrated interface circuit for piezoelectric harvesters. IEEE J. Solid State Circuits.

[28] Khan M.B., Kim D.H., Han J.H., Saif H., Lee H., Lee Y., Kim M., Jang E., Hong S.K., Joe D.J. (2019). Performance improvement of flexible piezoelectric energy harvester for irregular human motion with energy extraction enhancement circuit. Nano Energy.

[29] Khan M.B., Saif H., Lee Y. A Piezoelectric Energy Harvesting Interface for Irregular High Voltage Input with Partial Electric Charge Extraction with 3.9× Extraction Improvement. Proceedings of the 2019 IEEE Asian Solid-State Circuits Conference.

[30] Kwon D., Rincon-Mora G.A. (2014). A single-inductor 0.35 μm CMOS energy-investing piezoelectric harvester. IEEE J. Solid State Circuits.

[31] Badel A., Guyomar D., Lefeuvre E., Richard C. (2005). Efficiency enhancement of a piezoelectric energy harvesting device in pulsed operation by synchronous charge inversion. J. Intell. Mater. Syst. Struct..

[32] Sanchez D.A., Leicht J., Jodka E., Fazel E., Manoli Y. A 4µW-to-1mW parallel-SSHI rectifier for piezoelectric energy harvesting of periodic and shock excitations with inductor sharing, cold start-up and up to 681% power extraction improvement. Proceedings of the 2016 IEEE International Solid-State Circuits Conference (ISSCC).

[33] Quelen A., Morel A., Gasnier P., Grezaud R., Monfray S., Pillonnet G. A 30nA quiescent 80nW-to-14mW power-range shock-optimized SECE-based piezoelectric harvesting interface with 420% harvested-energy improvement. Proceedings of the Digest of Technical Papers-IEEE International Solid-State Circuits Conference.

[34] Lefeuvre E., Badel A., Richard C., Guyomar D. (2005). Piezoelectric energy harvesting device optimization by synchronous electric charge extraction. J. Intell. Mater. Syst. Struct..

[35] Meng M., Ibrahim A., Xue T., Yeo H.G., Wang D., Roundy S., Trolier-McKinstry S., Kiani M. Multi-Beam Shared-Inductor Reconfigurable Voltage/SECE-Mode Piezoelectric Energy Harvesting of Multi-Axial Human Motion. Proceedings of the 2019 IEEE International Solid-State Circuits Conference-(ISSCC).

[36] Cai Y., Manoli Y. A piezoelectric energy harvester interface circuit with adaptive conjugate impedance matching, self-startup and 71% broader bandwidth. Proceedings of the ESSCIRC 2017-43rd IEEE European Solid State Circuits Conference.

[37] Saif H., Khan M.B., Lee J., Lee K., Lee Y. (2019). A High-Voltage Energy-Harvesting Interface for Irregular Kinetic Energy Harvesting in IoT Systems with 1365% Improvement Using All-NMOS Power Switches and Ultra-low Quiescent Current Controller. Sensors.

[38] Hwang G.-T., Byun M., Jeong C.K., Lee K.J. (2015). Flexible Piezoelectric Thin-Film Energy Harvesters and Nanosensors for Biomedical Applications. Adv. Healthc. Mater..

[39] Park K.I., Son J.H., Hwang G.T., Jeong C.K., Ryu J., Koo M., Choi I., Lee S.H., Byun M., Wang Z.L. (2014). Highly-efficient, flexible piezoelectric PZT thin film nanogenerator on plastic substrates. Adv. Mater..

[40] Park I., Maeng J., Shim M., Jeong J., Kim C. A Bidirectional High-Voltage Dual-Input Buck Converter for Triboelectric Energy-Harvesting Interface Achieving 70.72% End-to-End Efficiency. Proceedings of the 2019 Symposium on VLSI Circuits.

[41] Chew Z.J., Zhu M. Combined power extraction with adaptive power management module for increased piezoelectric energy harvesting to power wireless sensor nodes. Proceedings of the Proceedings of IEEE Sensors.

[42] Saif H., Lee Y., Kim M., Lee H., Khan M.B., Lee Y. A wide load and voltage range switched-capacitor DC-DC converter with load-dependent configurability for DVS implementation in miniature sensors. Proceedings of the 2017 IEEE Asian Solid-State Circuits Conference.

[43] Razavi B. (2001). Design of Analog CMOS Integrated Circuits.

[44] Khan M.B., Saif H., Lee Y. (2020). A Piezoelectric Harvesting Interface with Capacitive Partial Electric Charge Extraction for Energy Harvesting from Irregular High-Voltage Input. Energies.

