# Supplementary material for: Dual Piezoelectric Energy Investing and Harvesting Interface for High-Voltage Input
Source: Sensors (Basel). 2021 Mar 28;21(7):2357. doi: 10.3390/s21072357 (PMC8037320; doi:10.3390/s21072357)
Supplement: Supplementary file 1 [file sensors-21-02357-s001.pdf]

sensors

MDPI

Article

Dual Piezoelectric Energy Investing and Harvesting Interface for High Voltage Input

Muhammad Bilawal Khan [1], Hassan Saif [2], Kyoungho Lee [3] and Yoonmyung Lee [1,*]

[1] Department of Electrical and Computer Engineering, Sungkyunkwan University, Suwon 16419, Korea; bilawal786@skku.edu
[2] Department of Electrical Engineering, National University of Computer and Emerging Sciences, Islamabad 44000, Pakistan; hassan.saif@nu.edu.pk
[3] Korea Electrotechnology Research Institute, Changwon 51543, Korea; khlee93@keri.re.kr
* Correspondence: yoonmyung@skku.edu

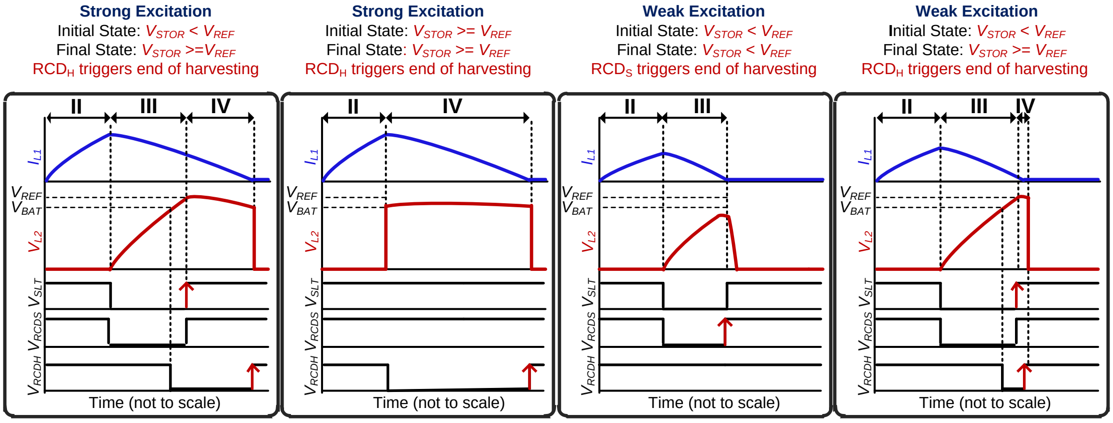

Figure S1. Conceptual waveform for the cases of harvesting and storage decisions made by HIC (a) Energy transferred to both C_{STOR} and battery, where SLT determines the end of energy transfer to C_{STOR}, (b) Energy transferred to battery only, (c) Energy transferred to C_{STOR} only, where RCD_S determines the end of energy transfer to C_{STOR}, (d) Energy transferred to both C_{STOR} and battery, where SLT determines the end of energy transfer to C_{STOR} and RCD_H immediately determines end of energy transfer to the battery.
